# Hospital epidemiology of emergent cervical necrotizing fasciitis

**DOI:** 10.4103/0974-2700.62108

**Published:** 2010

**Authors:** Nissar Shaikh, Firdous Ummunissa, Yolande Hanssen, Hussam Al Makki, Hamdy M Shokr

**Affiliations:** Departments of Anesthesia & ICU, Hamad Medical Corporation, Doha, Qatar; 1 Department of OBGY, Hamad Medical Corporation, Doha, Qatar; 2 Department of Clinical Pharmacy, Hamad Medical Corporation, Doha, Qatar; 3 Department of ENT, Hamad Medical Corporation, Doha, Qatar

**Keywords:** Cervical, diabetes mellitus, fresh frozen plasma, necrotizing fasciitis, packed red blood cells

## Abstract

**Background:**

Necrotizing fasciitis (NF) is a surgical emergency. It is a rapidly progressing infection of the fascia and subcutaneous tissue and could be fatal if not diagnosed early and treated properly. NF is common in the groin, abdomen, and extremities but rare in the neck and the head. Cervical necrotizing fasciitis (CNF) is an aggressive infection of the neck and the head, with devastating complications such as airway obstruction, pneumonia, pulmonary abscess, jugular venous thrombophlebitis, mediastinitis, and septic shock associated with high mortality.

**Aim:**

To assess the presentation, comorbidities, type of infection, severity of disease, and intensive care outcome of CNF.

**Methods:**

Medical records of the patients treated for NF in the surgical intensive care unit (SICU) from January 1995 to February 2005 were reviewed retrospectively.

**Results:**

Out of 94 patients with NF, 5 (5.3%) had CNF. Four patients were male. The mean age of our patients was 41.2 ± 14.8 years. Sixty percent of patients had an operative procedure as the predisposing factor and 80% of patients received nonsteroidal anti-inflammatory drugs (NSAIDs). The only comorbidity associated was diabetes mellitus (DM) in 3 patients (60%). Sixty percent of the cases had type1 NF. Mean sequential organ failure assessment (SOFA) score on admission to the ICU was 8.8 ± 3.6. All patients had undergone debridement at least two times. During the initial 24 h our patients received 5.8 ± 3.0 l of fluid, 2.0 ± 1.4 units of packed red blood cells (PRBC), 4.8 ± 3.6 units of fresh frozen plasma (FFP), and 3.0 ± 4.5 units of platelet concentrate. The mean number of days patients were intubated was 5.2 ± 5.1 days and the mean ICU stay was 6.4 ± 5.2 days. Sixty percent of cases had multiorgan dysfunction (MODS) and one patient died, resulting in a mortality rate of 20%.

**Conclusion:**

According to our study, CNF represents around 5% of NF patients. CNF was higher among male patients and in patients with history NSAIDs and dental surgeries. Type 1 NF was more common and DM was the only comorbid condition seen in this limited number of patients. The low mortality may be due to the early diagnosis and aggressive surgical treatment combined with optimal supportive intensive care management.

## INTRODUCTION

Necrotizing fasciitis (NF) is an abrupt-onset, rapidly progressive infectious process of fascia and subcutaneous tissue. NF can be fatal if not diagnosed early and treated aggressively. NF was first described in China by Meleny in 1924. Previously known as hospital gangrene, it was termed necrotising faciitis by Wilson in 1952. Suppurative fasciitis and necrotizing erysipelas are synonyms for NF.[1] The disease is characterized by fulminant destruction of tissue, local microvascular thrombosis, and systemic signs of toxicity. It is associated with high morbidity and mortality. NF is common in the groin, abdomen, and extremities but rare in the head and neck area. Cervical necrotizing fasciitis (CNF) is defined as an inflammation of the submandibular space with little or no suppuration. It tends to spread to the neck, involving more than one neck space. There is tissue necrosis with putrid infiltration, involvement of connective tissue and fascia, and secondary involvement of skin and muscles.[2] CNF is an aggressive infection of the head and neck with devastating complications such as airway obstruction, pneumonia, pulmonary abscess, jugular venous thrombophlebitis, mediastinitis, and septic shock. The mortality ranges from 12% to 30%.[2]

CNF can result from a breach in the integrity of mucosal membrane after trauma, surgery, or instrumentation, or it may follow odentogenic infections, tonsillitis, and peritonsillar abscess.[2]

The aim of this study was to assess the common comorbidities, the type of infection, the severity of the disease, first 24 hrs patient received Fluid, blood, blood products, and intensive care outcome of CNF.

## METHODS

Hamad Medical Corporation (HMC) at Doha, Qatar, is a university teaching hospital with a total capacity of 1600 beds. Surgical intensive care patients are admitted to Hamad General Hospital. The patient population includes locals as well as expatriates, mainly from other Arab countries and Asia. Healthcare (including medication) is free for locals and highly subsidized for other residents.

The medical records of all patients treated for NF between January 1995 to February 2005 in the surgical intensive care unit (SICU) of HMC were retrospectively reviewed. Only those patients in whom the diagnosis was confirmed by histopathology were included in the study. Ninety-four patients with NF were treated in the SICU during the study period and out of them five patients suffered CNF (5.3%).

The variables that were examined in this study included age, gender, predisposing factors, comorbid conditions, duration of symptoms, severity of disease, number of surgical debridement, initial resuscitative management, type of infection, duration of ICU stay, and patient outcome.

Statistical analysis was performed using the Statistical Package for Social Sciences (SPSS 16.0). Values are expressed as percentages, mean ± SD, or range.

The research committee of our hospital granted permission for this study.

## RESULTS

Ninety-four patients with NF were treated at the SICU. Five patients had cervical NF (5.53%). The mean age of our patients was 41.2 ± 14.8 years. Eighty percent of our patients were male. The only comorbid disease we found in our study sample was diabetes mellitus (60%). Sixty percent of patients had some dental operative procedure as the apparent predisposing factor and 80% of patients had received non-steroidal anti-inflammatory drugs (NSAIDs). The only comorbid disease was diabetes mellitus (DM), which was seen in three patients (60%). Sixty percent of the cases had type1 (polymicrobial) NF [Table 1]. All patients were febrile and had leucocytosis at the time of admission to the hospital [Table 2]. The mean number of debridements was 3.0 ± 1.0. Clinical parameters reflecting the severity of the condition – the mean sequential organ failure assessment (SOFA) score at admission to ICU – was 8.8 ± 3.6. All patients had undergone debridement at least two times. The duration of symptoms was 7.8 ± 8.2 days. During the initial 24 h our patients received 5.8 ± 3.0 l of fluid, 2.0 ± 1.4 units packed red blood cell (PRBC), 4.8 ± 3.6 units fresh frozen plasma (FFP), and 3.0 ± 4.5 units of platelet concentrate. The mean number of days for which patients were intubated was 5.2 ± 5.1 days and mean ICU stay was 6.4 ± 5.2 days [Table 2]. Sixty percent patient had multiorgan dysfunction syndrome (MODS) and one patient died, giving mortality of 20% in our study [Table 1].

**Table 1:** Demographic data, comorbid conditions, predisposing factors, type of CNF, frequency of MODS, and outcome

Parameter	Distribution of CNF patients (%)
Age groups	
<45	3 (60)
45-59	1 (20)
≥60	1 (20)
	
Gender	
Male	4 (80)
Female	1 (20)
Comorbid conditions	
NIDDM	3 (60)
Predisposing factors	
History of dental operation	3 (60)
History of trauma	0
History of insect bite	0
History of NSAID use	4 (80)
Type of CNF	
Type 1	3 (60)
Type 2	2 (40)
	
MODS	
Yes	3 (60)
No	2 (40)
Outcome	
Died	1 (20)
Survived	4 (80)

CNF: CERVICAL NECROTIZING FASCIITIS; NIDDM: NON-INSULIN-DEPENDENT DIABETES MELLITUS; NSAID: NONSTEROIDAL ANTI-INFLAMMATORY DRUGS; MODS: MULTIPLE ORGAN FAILURE SYDROME.

**Table 2:** Clinical parameters reflecting severity of condition while being in ICU

Parameter	Mean ± SD
WBC on admission	17.1 ± 7.0 × 103 /ul
Temperature (°C)	38.2 ± 1.3
Number of debridements	2.0 ± 1.0
SOFA score	8.8 ± 3.6
Duration of symptoms (days)	7.8 ± 8.2
Fluid required for 1^st^ 24 h	5.8 ± 3.0
Number of PRBC received for 1^st^ 24 h	2.0 ± 1.4
Number of FFP received for 1^st^ 24 h	4.8 ± 3.6
Number of platelets received for 1^st^ 24 h	3.0 ± 4.5
Intubated (days)	5.2 ± 5.1
ICU stay (days)	6.4 ± 5.2

FFP: FRESH FROZEN PLASMA; ICU: INTENSIVE CARE UNIT; PRBC: PACKED RED BLOOD CELL; SOFA: SEQUENTIAL ORGAN FAILURE ASSESSMENT; WBC: WHITE BLOOD CELL COUNT.

## DISCUSSION

In this study 80% of the patients presenting with CNF had used NSAIDs and 60% had a history of a dental surgical procedure as the predisposing factor. Helmy *et al*. also found in their study that the major etiological risk factor was dental surgery.[3] Male patients (60%) outnumbered the female patients, similar to the findings of a previous study where NF was more common in male patients.[3] The various predisposing factors for NF include DM, obesity, metastatic neoplasm, end-stage renal disease, hypothyroidism, drug abuse, and use of steroids.[4] DM was the only comorbid disease seen in three (60%) of our patients. High glucose levels form a good culture media for bacteria and predispose to an environment of low oxygen tension.[5] All our patients were febrile and had leucocytosis at the time of admission to hospital, which indicates the systemic manifestations of this devastating soft tissue infection. Recently published laboratory indicators for diagnosing NF score includes leucocytosis. By using this score, NF can be diagnosed early.[6] The majority of our patients received NSAIDs, which masks the manifestations even though the disease may be progressing. This masking effect can lead to delayed presentation as well as delayed diagnosis and management.[7] All our patients were admitted to SICU either before or right after debridement. These patients need aggressive resuscitation as large volumes of fluid sequestrate in the edematous wound. Capillary leak and significant hemorrhage may occur and many patients may suffer coagulation disorders.[4,8]

NF is microbiologically divided into two types: type 1 is polymicrobial and type 2 is monobacterial. Sixty percent of our patients presented with type1 NF. As mentioned in previous studies, type 1 infection is most common in CNF.[1] Skitarelic *et al*. however reported that type 2 NF was more common in their study, where the majority of CNF originated from a peritonsilar abscesses.[9] The second reason for type1 infection may be the predominance of DM in our patients.[10] Sixty percent of our patients had MODS and one of these patients died, giving mortality rate of 20%. This correlates with literature data from the early eighties, where one study reported a mortality of 22% in patients with NF of the head and the neck region.[11] A recent study however showed a higher mortality rate of up to 36% in patients with CNF, this despite all possible interventions, including hyperbaric oxygen therapy. The higher mortality in these patients was mostly due to delayed presentation and MODS.[3] We believe that the lower mortality rate seen in our patients was due to the combination of early aggressive surgical debridement and resuscitation in the SICU. This is in line with the recommendations made by Zilberstein *et al*.[2] and Helmy *et al*.[3] who suggested these measures for ensuring a better outcome for patients with NF.

The retrospective nature and the low power of our study are major limitations. We had only five patients with CNF in 10 years, which illustrates the rareness of this disorder. Because of the small sample size, statistical significance could not be established. Large prospective multicenter studies are needed but, because of the rarity of CNF, this is a challenge.

## CONCLUSION

In our study of 94 patients with NF, 5 patients had CNF. CNF was more common in male patients as well as in patients using NSAIDs and those having dental surgeries. In contrast to other reports, type 1 NF was more common. DM was the only comorbid condition detected. The low mortality rate in this sample is, in our opinion, most likely due to early diagnosis, aggressive surgical treatment, and good supportive intensive care management.
